# Digital endpoints in clinical trials: emerging themes from a multi-stakeholder Knowledge Exchange event

**DOI:** 10.1186/s13063-024-08356-7

**Published:** 2024-08-03

**Authors:** Mia S. Tackney, Amber Steele, Joseph Newman, Marie-Christine Fritzsche, Federica Lucivero, Zarnie Khadjesari, Jennifer Lynch, Rosemary A. Abbott, Vicki S. Barber, James R. Carpenter, Bethan Copsey, Elin H. Davies, William G. Dixon, Lisa Fox, Javier González, Jessica Griffiths, Chloe H. L. Hinchliffe, Magdalena A. Kolanko, Dylan McGagh, Aryelly Rodriguez, George Roussos, Karen B. E. So, Louise Stanton, Mark Toshner, Frances Varian, Paula R. Williamson, Belay B. Yimer, Sofía S. Villar

**Affiliations:** 1 MRC-Biostatistics Unit, University of Cambridge, Cambridge, UK; 2 Strategic Funding Partnerships Hub (SFPH), Cambridge University Hospitals, Cambridge, UK; 3 Department of Medicine, University of Cambridge and Royal Papworth Hospital, Cambridge, UK; 4 Institute of History and Ethics in Medicine, TUM School of Medicine and Health, Technical University of Munich, Munich, Germany; 5 School of Social Sciences and Technology, Technical University of Munich, Munich, Germany; 6 Ethox Centre, Nuffield Department of Population Health, University of Oxford, Oxford, UK; 7 School of Health Sciences, University of East Anglia, Norwich, England; 8 University of Hertfordshire, Hatfield, UK; 9 ICON PLC, Reading, UK; 10 Oxford Clinical Trials Research Unit (OCTRU), University of Oxford, Oxford, UK; 11 MRC Clinical Trials Unit at University College London, London, UK; 12 Department of Medical Statistics, London School of Hygiene and Tropical Medicine, London, UK; 13 Leeds Clinical Trials Research Unit, University of Leeds, Leeds, UK; 14 Aparito, a wholly owned subsidiary company of Eli Lilly and Company, Wrexham, Wales UK; 15 Centre for Epidemiology Versus Arthritis, Manchester Academic Health Sciences Centre, University of Manchester, Manchester, UK; 16 Clinical Trials and Statistics Unit (ICR-CTSU), The Institute of Cancer Research, London, UK; 17 Microsoft Research Cambridge, Cambridge, UK; 18 Translational and Clinical Research Institute, Newcastle University, Newcastle, UK; 19 UK Dementia Research Institute Care Research and Technology Centre, London, UK; 20 Imperial College London, London, UK; 21 Nuffield Department of Orthopaedics, Rheumatology and Musculoskeletal Sciences, University of Oxford, Oxford, UK; 22 Big Data Institute, Li Ka Shing Centre for Health Information and Discovery, University of Oxford, Oxford, UK; 23 The University of Edinburgh, Edinburgh, UK; 24 School of Computing and Mathematical Sciences, Birkbeck College, University of London, London, UK; 25 Alexion Rare Oncology, AstraZeneca, Cambridge, UK; 26 Southampton Clinical Trials Unit, University of Southampton, Southampton, UK; 27 Royal Papworth Hospital and Department of Medicine, Victor Phillip Dahdaleh Heart and Lung Research Institute, University of Cambridge, Cambridge, UK; 28 University of Sheffield, Sheffield, UK; 29 Institute of Population Health, University of Liverpool, Liverpool, UK; 30 Centre for Epidemiology, University of Manchester, Manchester, UK

**Keywords:** Digital endpoints, Digital health technology, Multi-stakeholder engagement, Clinical trials

## Abstract

**Background:**

Digital technologies, such as wearable devices and smartphone applications (apps), can enable the decentralisation of clinical trials by measuring endpoints in people’s chosen locations rather than in traditional clinical settings. Digital endpoints can allow high-frequency and sensitive measurements of health outcomes compared to visit-based endpoints which provide an episodic snapshot of a person’s health. However, there are underexplored challenges in this emerging space that require interdisciplinary and cross-sector collaboration. A multi-stakeholder Knowledge Exchange event was organised to facilitate conversations across silos within this research ecosystem.

**Methods:**

A survey was sent to an initial list of stakeholders to identify potential discussion topics. Additional stakeholders were identified through iterative discussions on perspectives that needed representation. Co-design meetings with attendees were held to discuss the scope, format and ethos of the event. The event itself featured a cross-disciplinary selection of talks, a panel discussion, small-group discussions facilitated via a rolling seating plan and audience participation via Slido. A transcript was generated from the day, which, together with the output from Slido, provided a record of the day’s discussions. Finally, meetings were held following the event to identify the key challenges for digital endpoints which emerged and reflections and recommendations for dissemination.

**Results:**

Several challenges for digital endpoints were identified in the following areas: patient adherence and acceptability; algorithms and software for devices; design, analysis and conduct of clinical trials with digital endpoints; the environmental impact of digital endpoints; and the need for ongoing ethical support. Learnings taken for next generation events include the need to include additional stakeholder perspectives, such as those of funders and regulators, and the need for additional resources and facilitation to allow patient and public contributors to engage meaningfully during the event.

**Conclusions:**

The event emphasised the importance of consortium building and highlighted the critical role that collaborative, multi-disciplinary, and cross-sector efforts play in driving innovation in research design and strategic partnership building moving forward. This necessitates enhanced recognition by funders to support multi-stakeholder projects with patient involvement, standardised terminology, and the utilisation of open-source software.

**Supplementary Information:**

The online version contains supplementary material available at 10.1186/s13063-024-08356-7.

## Background

### Engagement and exchange in the context of digital transformation

The digital transformation of health and social care services is now a key priority across healthcare systems and wider government infrastructure [1, 2]. Enabling digital innovation in the conduct of clinical trials to streamline the discovery and delivery of new interventions requires collaboration among several stakeholders, including healthcare providers, technology experts, researchers, regulators, policymakers and the public. Knowledge exchange activities and processes are crucial for fostering the generation and sharing of knowledge among diverse stakeholders from various sectors and disciplines and propelling progress [3]. While there is an emerging body of evidence in the implementation and evaluation of these activities in health care [4–6] and digital health [7–9], there is a need to highlight case examples of knowledge exchange activities so that learnings for effective practice can be shared. This article describes a Knowledge Exchange event on Digital Endpoints, outlining how the event was organised and facilitated, key themes that were discussed, and reflections and recommendations for future multi-stakeholder events.

### Digital endpoints as focus for Knowledge Exchange

New interventions are tested in clinical trials to evaluate whether they have a specific effect on patients’ health outcomes. These health outcomes are called endpoints. Digital endpoints are novel endpoints that are measured using digital technologies such as wearable devices or smartphone applications (apps) and do not require assessment in a clinical setting [10]. Examples include digital walk tests to measure exercise capacity [11], physical activity measures captured via wrist-worn accelerometers [12], electronic patient-reported outcomes completed via apps [13] and digital assessments of motor symptom severity [14]. Digital endpoints offer opportunities to change the quantity and quality of data collection and can improve patient experiences in trials. Compared to episodic in-clinic assessments such as the 6 Minute Walk test or polysomnography, endpoints captured by digital technologies typically allow for substantially more frequent measurement of health outcomes and in an individual’s chosen location(s) (instead of at the clinic) [15]. This is an example of a decentralised component of a trial, where the trial activity (in this case, data collection) occurs at locations other than traditional clinical trial sites [16]. Digital endpoints can reduce burden on patients and their carers and the captured data may more realistically reflect individuals’ experiences [17] and may reduce financial costs [18] and environmental impacts of trials [19]. There have been key developments from regulators for digital endpoints, such as the approval of Stride Velocity 95th Centile by the European Medicine Agency (EMA) as a primary endpoint for Duchenne Muscular Dystrophy in pivotal or exploratory drug therapeutic studies [20], and development of guidelines on the use of Digital Health Technologies in clinical investigations by the Food and Drug Administration (FDA) [21].

There are several challenges to widespread adoption of digital endpoints. Known challenges include questions around privacy and potential to exacerbate unequal access to digital technology [22, 23], the limited number of regulatory-approved devices and digital endpoints (for pivotal and licensing trials), and lack of unified terminology around digital endpoints [24]. Since addressing such challenges requires diverse expertise and a range of stakeholder perspectives, interdisciplinary collaborations have been called for [10, 25, 26]. Key perspectives in the ecosystem for the development, validation, and use of digital endpoints include clinicians, patients, statisticians, computer scientists, ethicists, regulators and health economists, among others. We refer to these perspectives as *stakeholder* perspectives and refer to communities which consist largely of one stakeholder perspective as a *silo* or *stakeholder silo*.

In November 2023, a Knowledge Exchange event on digital endpoints was organised in Cambridge, UK. This event, featuring a co-design process with session speakers, aimed to facilitate collaboration within and between stakeholder silos for a more comprehensive understanding of emerging challenges for digital endpoints.

## Methods

### Curation of attendee list

The curation of the attendee list was an iterative process which took several months. Organisers sent an initial survey to key stakeholders within their network, asking for ideas for potential talks and discussion topics. From this core group, organisers identified stakeholder perspectives that needed representation and extended invitations to individuals who could represent these perspectives. Discussions often led to the discovery of additional stakeholder perspectives that the organisers had not originally considered. These included perspectives on Core Outcome Sets, which are an agreed standard set of outcomes that should be measured and reported, as a minimum, in trials for a specific area of health or health care [27], and greener research practices for digital endpoints.

To include the patient and public perspective at the event, details of the event were circulated in a newsletter for the Cambridge University Hospital Patient and Public and Public Involvement (PPI) Panel (two attendees participated). A clinician attending the event invited participants from his ongoing Pulmonary Hypertension studies. One participant attended the event and two participants provided feedback which were integrated within the clinician’s presentation in the form of a video and comments. An additional patient representative was invited through connections with the investigator of a hypoglycaemia study which involved continuous glucose monitoring.

Organisers endeavoured to keep the size of the group relatively small and aimed for approximately 30 attendees so that discussions could be meaningful.

### Co-design meetings

Prior to the event, organisers held co-design meetings with speakers in each session to discuss speakers’ perspectives on key challenges and the format of the session. These co-design sessions emphasised the importance of sharing opinions and experiential knowledge during the event and encouraged speakers to focus their talks on questions such as: What keeps you up at night when it comes to digital endpoints? What are your concerns around digital endpoints that you would like other stakeholders to realise? The co-design sessions allowed for networking to take place before the event and helped to set intentions for an open and collaborative atmosphere.

### Event structure

The event was structured into four sessions, as detailed in Table A1 in the Supplementary Information. The first three sessions featured three short talks followed by small-group sessions. The last session included a keynote talk and a panel-led discussion.

**Table 1:** Details of Knowledge Exchange event

Name	Knowledge Exchange Event on Digital Endpoints
Date	29th November 2023
Venue	MRC-Biostatistics Unit, University of Cambridge
Organisers	Mia Tackney (Research Associate, MRC-Biostatistics Unit) Sofía Villar (Programme Leader, MRC-Biostatistics Unit) Amber Steele (Senior Research Advisor, Research Support Service)
Number of participants	32 in-person attendees. Five individuals contributed remotely (by providing a recorded video or comments)
Funding	All Council Harmonised IAA Rapid Response Award, NIHR Cambridge Biomedical Research Centre (NIHR203312) and Cambridge Centre for Data-Driven Discovery
Payment/compensation	All attendees were reimbursed for travel/accommodation expenses if required. PPI contributors were offered payment for their time

Organisers facilitated interdisciplinary discussions through rolling seating arrangements and the use of technology. In the first session, attendees were grouped into discussion tables corresponding to stakeholder silos. This allowed them to reflect on the talks from their specific stakeholder perspective. In the second and third sessions, attendees were grouped into cross-sector and cross-disciplinary tables to facilitate networking and exchange across silos. The interactive platform, Slido, was used throughout the day. It collated information from the whole group in the form of word clouds (see Figs. A1 and A2 in the Supplementary Information) and facilitated collection of questions and reflections from discussion tables. Questions and reflections were projected on the screen, which allowed attendees to see topics discussed in tables other than their own. A selection of questions was posed to the panel as discussion questions at the end of the day.

Table 1 provides key details of the Knowledge Exchange event.

### Feedback processes

After the event, a feedback form was sent to attendees which queried whether the event led to exchange of stakeholder perspectives, and invited comments on the programme, topics and perspectives represented and the format of the event. Patient and public contributors were invited to provide feedback in an online meeting. Two contributors attended the meeting and summarised their feedback in a short text.

#### Theme extraction

A transcript was generated from an audio recording of the event, which captured the talks and plenaries. The transcript and the summarised reflections and questions from small group discussions on Slido were used to identify key themes. This was achieved by an iterative process where one author grouped emerging themes in a summary table. The organisers and a selected cross-sector group of attendees discussed the summary table and also identified the scope, aims and target audience of the summary article. All attendees were invited to be co-authors of the paper and the selected themes were shared with all co-authors and revised based on feedback.

## Results

### Attendee representation

**Fig. 1:**
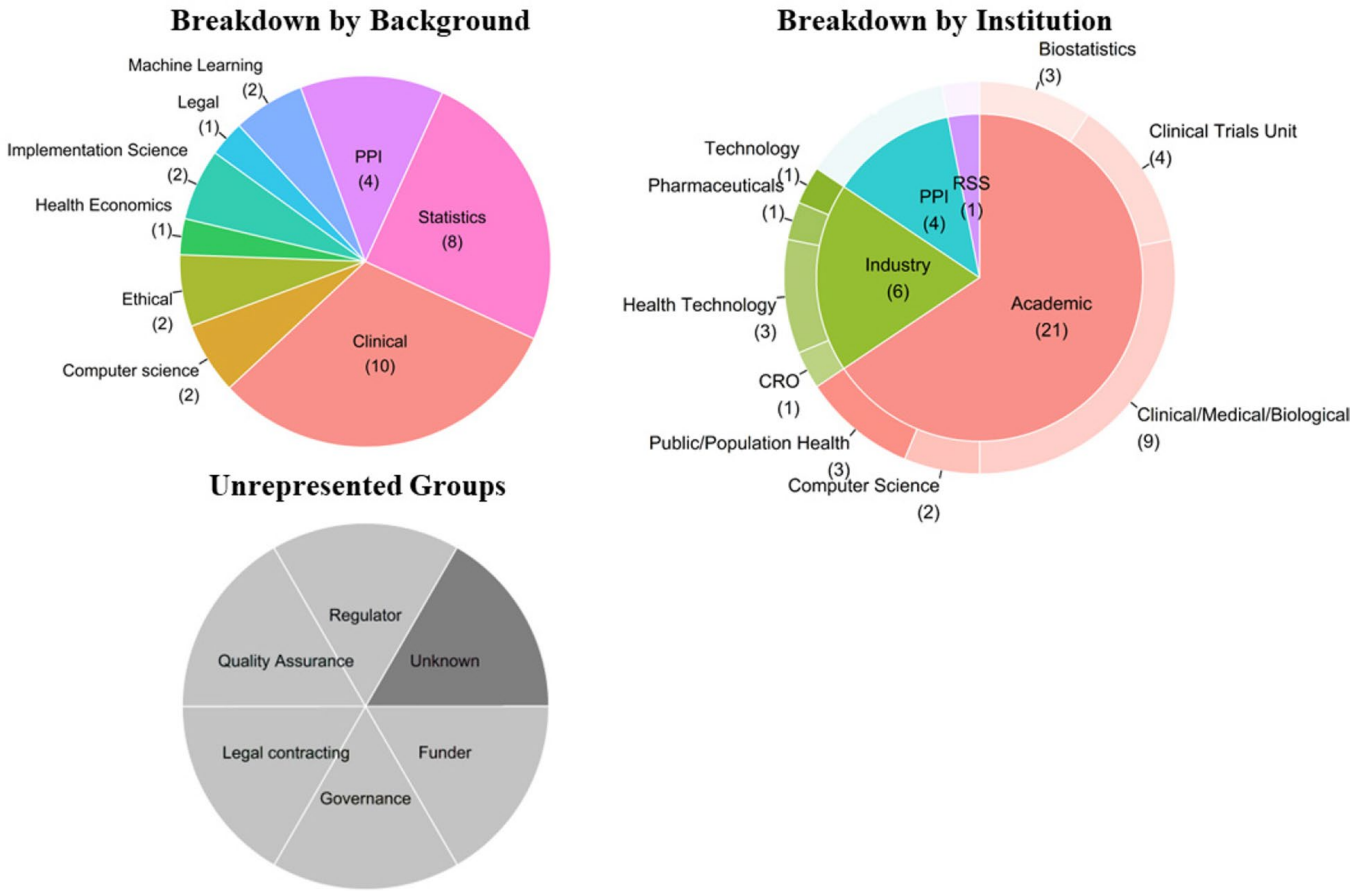
Breakdown of the number of in-person attendees at the event by background (top left) and by institution (top right). Perspectives that were not represented in the event, and would be important to have representation for future events, are indicated in the grey pie chart (bottom left). PPI, Patient and Public Involvement; RDS, National Institute of Health and Care Research (NIHR) Research Support Service; CRO, Clinical Research Organization

In-person attendees at the event included 32 individuals from diverse expertise backgrounds representing key silos across the ecosystem with interest and/or experience in digital endpoints, including clinicians, statisticians, computer scientists, implementation scientists, ethicists, health economists and patient and public representatives. Their experiences with digital endpoints spanned several disease areas, including pulmonary hypertension, dementia, Parkinsons’, women’s health and diabetes. Figure 1 illustrates attendee perspectives in terms of their background and sectors/institution, and also indicates some perspectives that were not represented on the day and would be important to have representation for future events.

### Themes

#### Patient adherence and acceptability

A key theme discussed was barriers to patient adherence and acceptability of digital endpoints. Digital endpoints typically require patients to participate in data collection for a long period of time in their everyday conditions and without the presence of clinical or research professionals. Obtaining data of high quality relies on acceptability of the digital approach to data collection. A PPI contributor who had participated in digital trials noted the convenience of doing assessments from home “without the upheaval of having to attend an appointment” but also highlighted technical challenges such as connectivity issues. Invited clinicians emphasised the need to consider specific patient needs in the continual process of designing and customising digital technologies and apps, for example providing a magnification tool to allow patients with poor eyesight to use the device.

Patient adherence in longer-term studies typically declines over time and can also depend on disease severity [28, 29]. Invited clinicians shared experiences of observing that patient adherence is lower at either extreme of disease severity. Individuals may engage less when the disease does not have much impact on their lives, and when the disease is severe, individuals may not be well enough to prioritise engaging with studies. An invited clinician also noted the impact of investigators’ interest in digital endpoint adoption and said: “investigator engagement I think sometimes [is] overlooked… I need to be able to demonstrate to a clinical colleague that what we are doing is validated enough, for example, that they are willing to accept and adopt it.” It was also highlighted that investigators' enthusiasm for adopting digital technology can lead to different levels of patient adherence across sites within a study. Strategies to help sustain engagement were discussed, including providing ongoing technical support to patients throughout the study, using nudges, notification and gamification strategies to enhance engagement, having an investigator in the loop, providing ongoing feedback to patients, and garnering support from communities and charities.

Implementation scientists highlighted that the “effectiveness of an intervention does not guarantee its uptake in routine use” and presented frameworks, e.g. non-adoption, abandonment, scale-up, spread, and sustainability (NASSS) framework [30], which can help researchers identify and assess the barriers and facilitators to adoption of digital technology in context. Implementation strategies can be used to tackle these barriers [31]; qualitative and quantitative approaches, including validated measures [32] can be used to ascertain the success of implementation outcomes, such as acceptability or adoption [32]. The discussion highlighted the scarcity of implementation science expertise in current trials research and the need to incorporate more of this expertise into research funding proposals.

#### Software and algorithms for devices

Digital endpoints which capture physiological characteristics typically use algorithms and software to convert sensor measurements into clinically meaningful outcomes. Several challenges around the validation of these algorithms and their use in specific study populations and contexts were highlighted. For example, researchers working on the validation of mobility and sleep-related outcomes discussed the complexities of validation in individuals’ free-living conditions. Compared to validation in lab settings, data from free-living conditions have increased inter-subject and within-subject variability. Further, they emphasised that important contextual factors may be unknown, such as whether an individual is typically in an area with open outdoor space or in an indoor space with obstructions [33], and these contextual factors can have an impact on mobility outcomes such as gait. Additional challenges included missing data, unequal representation of different groups in the data, and unexpected issues such as devices worn incorrectly.

There are also difficulties with using readily-developed algorithms for digital outcomes. Such algorithms are typically validated on one population and may not be suited for use in other populations. For example, researchers from Clinical Trials Units discussed the challenges of using thresholds based on healthy populations to quantify outcomes such as time spent in sleep using activity monitors in a trial for stroke recovery. The majority of validation studies are conducted in healthy populations and information on thresholds for activity monitoring data in chronic disease populations is lacking [34]. There is a need for further research on how to adapt these thresholds for specific populations, such as the work by Airlie et al. (2022) on adapting minimal wear time criteria in older care home residents [35].

An important discussion point was that using proprietary software has a disadvantage that the underlying algorithms are unknown to the researcher, and can also be changed by the developers unbeknownst to the researcher. Making explicit, for example in the contract between researchers and private companies, to communicate any changes in software was discussed, and the benefits of devices that allow extraction of raw data and open source software were also highlighted.

#### Design, analysis and conduct of clinical trials with digital endpoints

Using digital endpoints to evaluate health interventions leads to several open questions in the design, analysis and conduct of clinical trials. A key discussion point was on the choice of digital endpoint, since there is currently vast heterogeneity in digital endpoints and lack of standards in how they should be selected and reported [17]. There was a discussion about the potential for digital endpoints to be included in core outcome sets (COS), which are an agreed standard set of outcomes that should be measured and reported, as a minimum, in trials for a specific area of health or health care [27]. Core outcomes should be determined through a rigorous consensus process that involves people with lived experience of the health condition, healthcare professionals who care for those people, and researchers who would use the COS in their studies. Digital technology may be a viable option for measuring a core outcome, provided agreed standards for validity, reliability, feasibility and acceptability are met [36].

There were discussions on aspects of the design and analysis of trials with digital endpoints which lack clear guidance. Questions arose about the appropriate duration of the measurement period for individuals engaging with digital devices, whether there should be a gap between baseline and follow-up measurements, and if so, how long this gap should be. Clarity is needed regarding a sufficient amount of time for data collection, as investigators may wish to collect as much data as possible. An invited statistician emphasised that “we need to make sure that what we’re doing is not measuring more than we need to measure.” There was discussion on whether the data collected over time should be summarised into a single measure, or whether the entire time series should be analysed through, for example, Generalised Additive Models (GAMs) [37]. Certain endpoints, such as those relating to physical activity, can be strongly influenced by weather and seasons, which can lead to confounding. For example, physical activity has been shown to increase with increased daylight hours [38] and reduce with rainfall [39]. Statisticians mentioned possible approaches to mitigate the impact of seasonal effects through the recruitment of the trial as well as in the statistical analysis [40]. Finally, handling missing data for digital endpoints was a key discussion point, as digital endpoints may have complex missing data patterns which include missing not at random (MNAR) mechanisms [41–43].

Current operational practices on management of data need adapting for digital endpoints. For example, an industry statistician noted that digital endpoints are typically received directly from the vendor and do not go through standard in-house data cleaning processes by data management typical of data entered at site. Therefore, there is a greater need to pre-specify potential outliers/abnormalities in the Statistical Analysis Plans (SAPs) as it is more likely that they are dealt with in the analysis stage (rather than by data management). Further, the Study Data Tabulation Model (SDTM) [44], a common approach to structuring trial datasets requires adjustment for digital endpoint data which is not visit-based and leads to high-frequency data over a longer period.

#### Environmental impact of digital endpoints

Representatives from the Medical Research Council National Institute of Health and Care Research (MRC NIHR) Trials Methodology Research Partnership Greener Trials group discussed how data collection for digital endpoints may impact the carbon footprint of a trial. While the carbon footprint may decrease due to reduced travel by participants and reduced use and shipment of paper-based assessments, there is a need to consider and quantify the environmental impact of the manufacture, use, transport and disposal of digital devices and the storage of large amounts of data. A guidance document was presented which quantifies the carbon footprint of clinical trial activities, including carbon footprinting of digital devices, online questionnaires and data linkage [45]. A reflection on Slido was posted stating that “reducing the carbon footprint [of clinical trials via de-centralisation] requires different stakeholders to work in synergy (methodologists, data analysts, implementation scientists, funders, trial managers, etc.).”

#### Ongoing ethical support

Invited ethicists emphasised the need to integrate ethical reflection throughout the duration of studies utilising digital technology. Ongoing ethical reflection, as opposed to simply at the initial institutional approval, is important for two reasons, among others. Firstly, Research Ethics Committees are currently not equipped to assess these studies in a consistent way [46], and secondly, the remote and patient-dependent nature of these studies necessitates ongoing assessment of ethical issues. These studies require patients to engage with digital tools and monitoring in their daily spaces, which entails some level of responsibilisation of patients for the success of the study. This also raises issues around the role of family members and issues around privacy of bystanders (for example, when wearable cameras are used to collect data). While some issues can be envisioned at the stage of protocol design, real-world contexts may introduce unforeseen behavioural, cultural, and moral challenges that research teams must address. Ongoing ethical support can be delivered by involving ethicists throughout the study from the stage of developing a project to the delivery and assessment of the study [47, 48]. This enables the research team to identify not only known ethical issues as they appear in the literature and mitigate them in the development of the study protocol, but also to anticipate issues that are specific to the study. “Ethics clubs or clinics”, as part of regular meetings for clinical or research teams provide opportunities to discuss emerging issues together with an ethicist and embed ongoing ethical support. Inclusion of ethicists in steering committees also ensures that ethical issues are addressed and acted upon not only from bottom-up (from the research practice) but also top-down (from the leadership team).

During the event, several issues were raised which required ethical reflection, including the type of feedback to provide patients regarding their data, and how Adverse Events and Serious Adverse Events identified by digital technology should be evaluated and managed.

Furthermore, issues of fairness were highlighted, such as the exclusion of certain groups from studies, and the risk of producing results that are skewed towards certain populations. It was also pointed out how researchers using third party devices are often dependent on tech companies’ extraction and interpretation of raw data without being able to access raw data and other relevant information. The power imbalance between public research organisations and big tech corporations is an ongoing issue in digital endpoint research.

#### Need for multi-stakeholder collaboration

The Knowledge Exchange event highlighted the need for greater opportunities for open-forum discussions between stakeholders and highlighted the importance of consortia for development in this area, such as Mobilise-D [49], the Clinical Trials Transformation Initiative (CTTI) [50] and the Digital Medicine Society (DiMe) [51]. From an academic perspective, a need for funding organisations to offer additional support, guidance and opportunities for funding cross-disciplinary and cross-sector collaboration projects was called for, as the existing system often prioritises single-discipline approaches [52]. The importance of early engagement with regulators was emphasised, as well as a need for academics and funders to understand the regulatory requirements for operating in clinical trials versus clinical care and the pathways required. During the panel discussion, having open forums where there is open dialogue, as well as open source software and standardised terminology were mentioned as important facilitators for digital endpoint development. Recent work by the European Federation of Pharmaceutical Industries and Associations [24] and the Digital Medicine Society [53] exemplifies efforts on developing harmonised terminology.

A summary of the discussed themes is provided in Table 2.

**Table 2:** Summary of key themes

Theme	Key topics and recognised needs
**Patient acceptance and acceptability**	Impact of disease severity and investigator enthusiasm on engagement
	Importance of integrating patient needs in the design of devices
	Inclusion of implementation science expertise
**Software and algorithms for devices**	Higher variability and unknown contextual factors when collecting data in daily lives
	Limitations of using proprietary software
	Validation of algorithms in new contexts if originally validated in healthy populations
**Design, analysis and conduct of clinical trials with digital endpoints**	Heterogeneity in digital endpoints and lack of standards
	Use of digital technology to measure core outcomes
	Need for guidance on issues such as seasonal variation and missing data
	Operational challenges for industry trials
**Environmental impact**	Quantification of carbon footprint for the manufacture, use, transport and disposal of digital devices
**Ongoing ethical support**	Introduction of new ethical issues related to digital data collection (unanticipated at protocol design stage)
	Mechanisms of support through e.g. Ethics clubs and involvement in steering committees
	Tackling power imbalance between researchers and big tech companies
**Need for multi-stakeholder cooperation**	Need for funding support for cross-disciplinary and cross-sector collaboration
	Importance of early engagement with regulators
	Use of open source software and standardised terminology

### Event feedback

Attendees noted in the feedback form that the event broadened their understanding of other stakeholder perspectives and disease areas and that these learnings would help inform their work. The rolling seating plan for enhanced networking, small-group discussions and the use of Slido were well-received. Organisers noted the challenge for speakers to communicate their message to a broad audience and recommended future events to undertake additional co-design work to support cross-sector and cross-disciplinary communication.

Additional feedback from attendees called for a presentation on the operational aspects of running digital trials, as well as more opportunities for the PPI perspective to be heard at the event, for example through a presentation given by a PPI representative.

### Learnings from PPI contributors

**Fig. 2:**
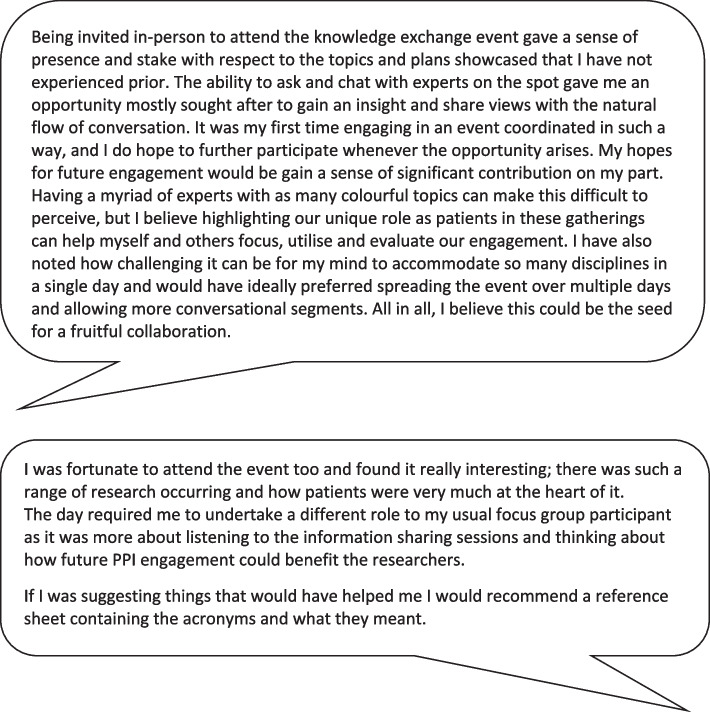
Perspectives of two PPI contributors

Feedback from PPI contributors showed considerable variation in their experience of the event and the extent to which they felt they could contribute. Some PPI contributors felt that there was too much jargon, and there was a lack of clarity on expectations about how they should contribute to the event, while other PPI contributors enjoyed the opportunity to engage with experts. PPI contributors provided ideas on how the event structure could be improved to facilitate more interaction between the PPI contributors and other stakeholders, which included providing a glossary of key terms to the PPI contributors, and making the event longer to allow more time for discussion. See Fig. 2 for quotes from two PPI contributors on their reflections. The event highlighted a need for better guidance on how to design multi-stakeholder events which allow PPI contributors to engage meaningfully [54, 55]. Additional ideas on facilitators to PPI contributions included providing a plain English summary explaining the purpose of a Knowledge Exchange event and plain English summaries for each of the talks.

## Discussion

### Key themes and under-explored challenges

The multi-stakeholder event highlighted emerging challenges and needs for digital endpoint development. This included: underexplored barriers to patient adherence, such as investigator enthusiasm towards digital endpoints, which was a novel insight uncovered during the event; the importance of implementation methods and ethical support throughout the entire lifecycle of research; the need for validation of algorithms in diverse populations and the importance of consortium building across disciplines and sectors.

### Limitations

Securing representation from certain stakeholder silos was challenging; for example, organisers were unable to find a regulator who was able to attend the event, but one attendee had previous experiences of working as a regulator, and several attendees had experiences of interacting with regulators.

There were several key stakeholders who were not represented on the day, which were noted during and after the event. These included individuals working within governance, legal contracting and regulatory bodies, whose perspectives were noted as important to involve in future events due to their role as gatekeepers and facilitators for progress in the digital transformation of trials. There also was no funder representative in the room. Given that the need for change in infrastructures and incentives to allow more collaborative and cross-sector work was a key theme and challenge that emerged, the lack of a funder perspective was noted by an invited clinician as a “missed opportunity” due to their key role in “shaping the landscape”.

There was also recognition that there were several important topics that were not discussed on the day. These include, and are not limited to: situations where digital endpoints should not, or cannot be used; the potential for increased digital divide due to digital endpoints; and the challenge of meeting different priorities for each of the stakeholders in the research ecosystem. Further, we note a number of comprehensive papers that cover the ethical topics around digital endpoints that provide a basis for consideration of topics that were not covered on the day and could guide next generation events [16, 56, 57].

Organisers also became aware of methodological frameworks to support multi-stakeholder facilitation [58, 59] after the event, and encourage consideration and use of these frameworks in next generation events.

## Conclusion

The Knowledge Exchange event demonstrated that, in the space of digital endpoints, there is appetite for dynamic processes of exchanging and sharing knowledge from multiple sources [60, 61]. The event serves as an example of a multi-stakeholder event with co-designed features to explore key and under-explored challenges. Several learnings were taken on topic clusters and stakeholder perspectives that need representation in future events, and learnings were generated through discussion with PPI contributors on changes to the organisation and provision of materials which could improve PPI contributors’ experience and ability to contribute meaningfully. These learnings provide useful considerations for next generation multi-stakeholder events.

### Supplementary Information

Supplementary Material 1: Table A1. Schedule of Knowledge Exchange Event. Figure A1. Attendees were asked at the start of the day to state what core perspective they were bringing to the event. Responses that are repeated are indicated by larger fonts/distinct colours. Figure A2. Attendees were asked at the start of the day what they thought were the key challenges with digital endpoints. Responses that are repeated are indicated by larger fonts/distinct colours.

## Data Availability

Not applicable.
