# Supplementary material for: Digital endpoints in clinical trials: emerging themes from a multi-stakeholder Knowledge Exchange event
Source: Trials. 2024 Aug 3;25:521. doi: 10.1186/s13063-024-08356-7 (PMC11297702; doi:10.1186/s13063-024-08356-7)
Supplement: Supplementary file 1 — Supplementary Material 1: Table A1. Schedule of Knowledge Exchange Event. Figure A1. Attendees were asked at the start of the day to state what core perspective they were bringing to the event. Responses that are repeated are indicated by larger fonts/distinct colours. Figure A2. Attendees were asked at the start of the day what they thought were the key challenges with digital endpoints. Responses that are repeated are indicated by larger fonts/distinct colours. [file 13063_2024_8356_MOESM1_ESM.docx]

# Multimedia Appendix

*Table A1: Schedule of Knowledge Exchange Event*

| Arrival and Coffee |  |
| --- | --- |
| Introduction: Why are we here, what’s your stakeholder hat, what are the differing perspectives on key challenges? |  |
| Session 1. Challenges in the uptake/use of digital technology: why context matters Chair: Mark Toshner (Victor Phillip Dahdaleh Heart & Lung Research Institute, University of Cambridge) |  |
| Speakers   - Joe Newman (University of Cambridge & Royal Papworth Hospital) *Engaging patients in the design, testing and use of digital endpoints: a clinical perspective* - Zarnie Khadjesari (University of East Anglia) *Why and how should we measure implementation success?* - Federica Lucivero (University of Oxford) *Ethical reflection before and during digital endpoints research: why and how*   Small group discussion: What question/thought do you have now, that you didn't have before you heard these talks as it relates to your stakeholder silo area. | |
| Coffee Break |  |
| Session 2. Challenges in Algorithms for Devices Chair: Cecilia Mascolo (Department of Computer Science and Technology, University of Cambridge) |  |
| Speakers   - Chloe Hinchcliffe (Newcastle University)  *Exploring the relationships between real-world gait and abnormal fatigue and sleepiness in chronic diseases* - Magda Kolanko (Imperial College London & Dementia Research Institute)  *Algorithms for digital (non-wearable) technology - continuous sleep monitoring in drug trials* - Dylan McGagh (University of Oxford)  *Utilising a hybrid self-supervised step detection model to explore associations between step count and cadence with inflammatory arthritis within the UK Biobank*   Small group discussion: What unique perspective/advice can you offer to round out the discussion of these challenges? | |
| Lunch |  |
| Session 3. Challenges in Statistical Methodology Chair: Mia Tackney (MRC-Biostatistics Unit, University of Cambridge) |  |
| - Rosemary Abbott (ICON PLC)  *Challenges with digital endpoints, from an industry statistical standpoint* - Bethan Copsey (Leeds CTU)  *Challenges with analysing data from wearables for the RECREATE stroke recovery trial: perspective from an academic trials unit* - Paula Williamson (University of Liverpool), Jessica Griffiths and Lisa Fox (The Institute of Cancer Research) *Relevance of core outcome sets and carbon footprinting of digital endpoints* [Recorded video]   Small group discussion: What unique perspective/advice can you offer to round out the discussion of these challenges? | |
| Coffee Break |  |
| Session 4. Challenges in Regulatory/legal/ethical areas Chair: Anne Blackwood (Health Tech Enterprise) |  |
| Speaker   - George Roussos (Birkbeck College, University of London)  *Towards regulatory acceptance of digital outcome measures for Parkinson’s disease*   Discussion facilitated by Anne Blackwood with panellists (20 min):   - George Roussos (Birkbeck College, University of London) - Elin Haf Davies (Aparito) - Federica Lucivero (University of Oxford) | |
| Closing Remarks |  |


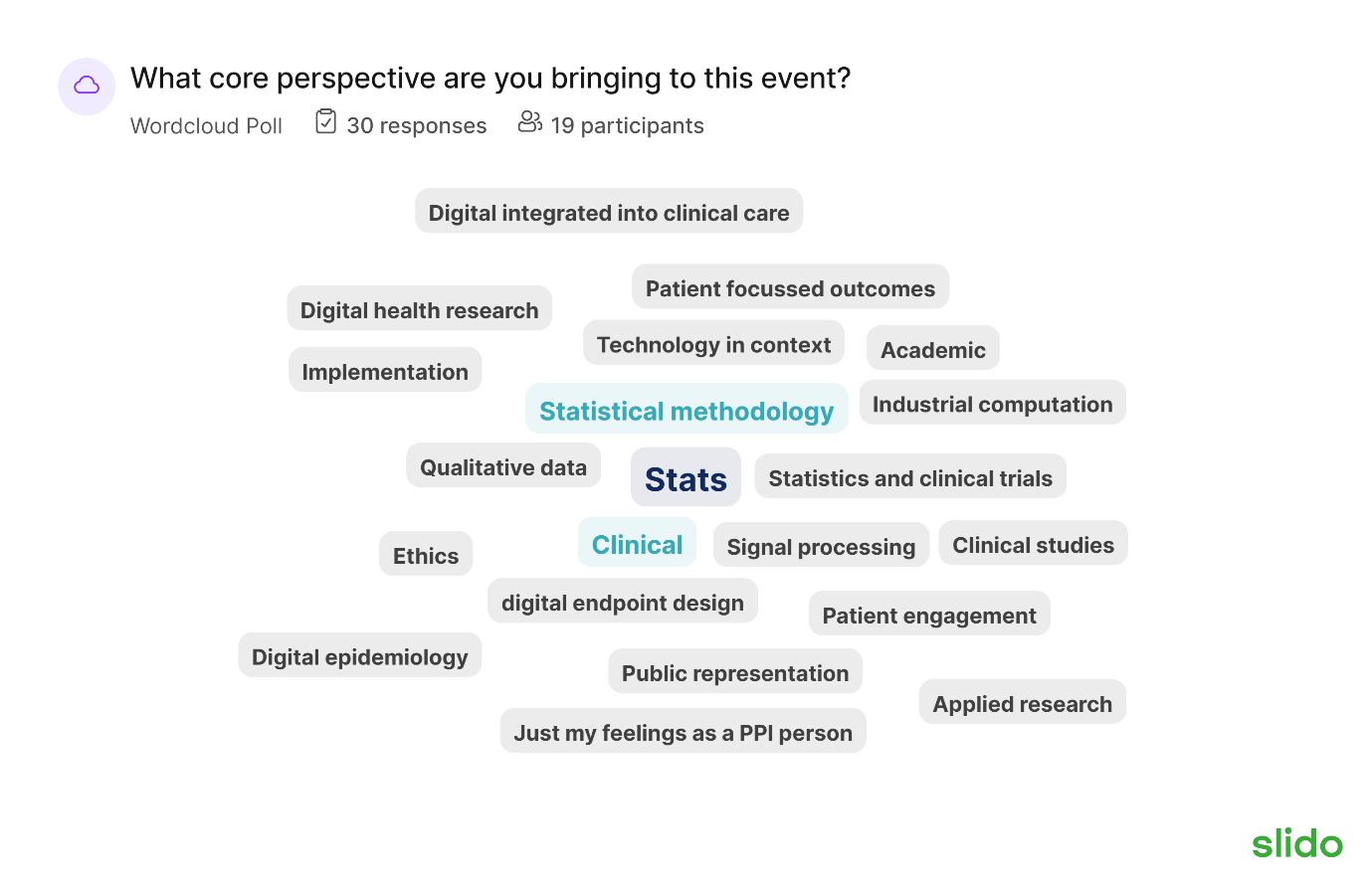


*Figure A1: Attendees were asked at the start of the day to state what core perspective they were bringing to the event. Responses that are repeated are indicated by larger fonts/distinct colours.*


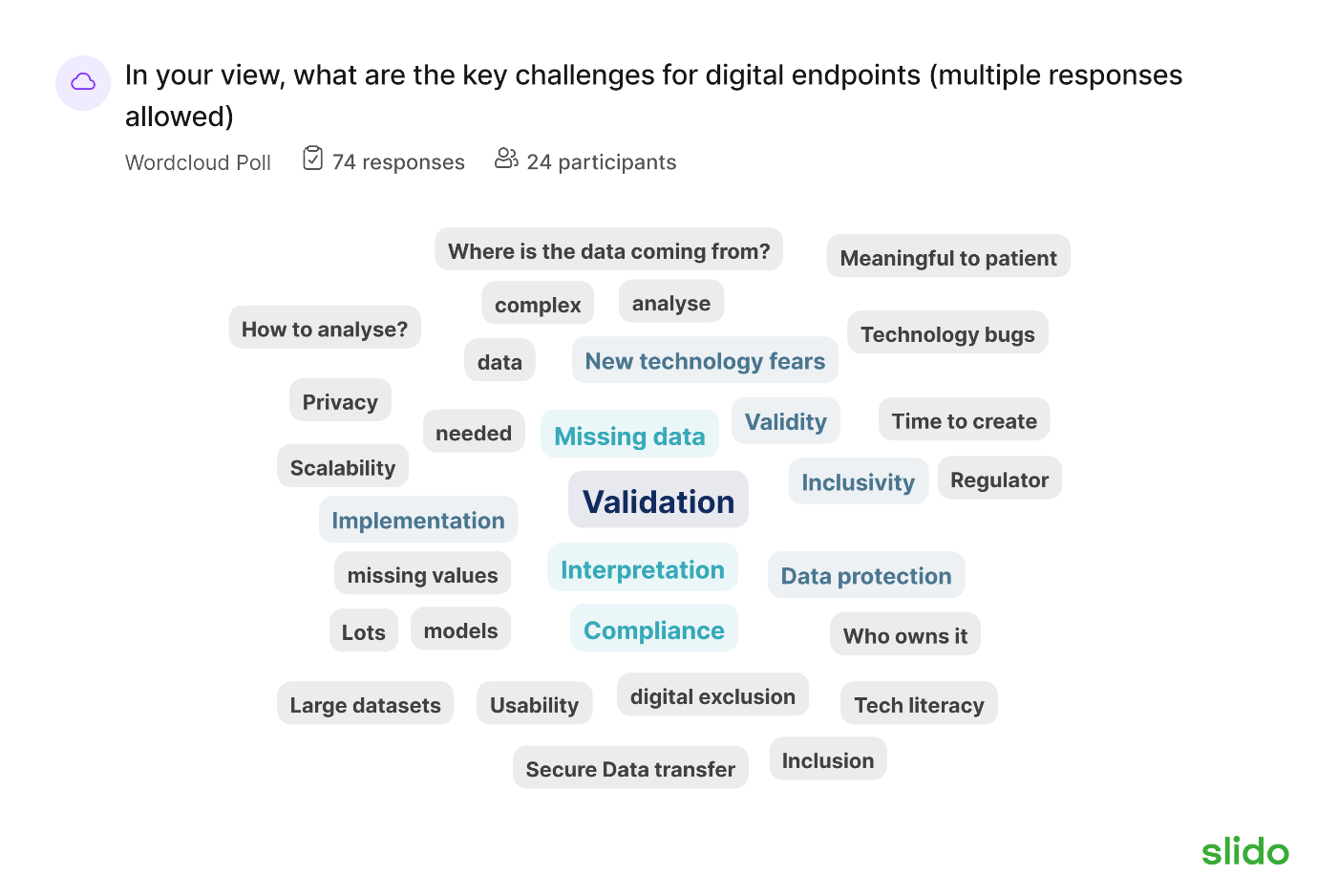


*Figure A2: Attendees were asked at the start of the day what they thought were the key challenges with digital endpoints. Responses that are repeated are indicated by larger fonts/distinct colours.*
